# RNAi of a Putative Grapevine Susceptibility Gene as a Possible Downy Mildew Control Strategy

**DOI:** 10.3389/fpls.2021.667319

**Published:** 2021-05-28

**Authors:** Demetrio Marcianò, Valentina Ricciardi, Elena Marone Fassolo, Alessandro Passera, Piero Attilio Bianco, Osvaldo Failla, Paola Casati, Giuliana Maddalena, Gabriella De Lorenzis, Silvia Laura Toffolatti

**Affiliations:** Dipartimento di Scienze Agrarie ed Ambientali, Università degli Studi di Milano, Milan, Italy

**Keywords:** susceptibility gene, gene silencing, dsRNA, obligate parasite, disease resistance, *Vitis vinifera*

## Abstract

Downy mildew, caused by the oomycete *Plasmopara viticola*, is one of the diseases causing the most severe economic losses to grapevine (*Vitis vinifera*) production. To date, the application of fungicides is the most efficient method to control the pathogen and the implementation of novel and sustainable disease control methods is a major challenge. RNA interference (RNAi) represents a novel biotechnological tool with a great potential for controlling fungal pathogens. Recently, a candidate susceptibility gene (*VviLBDIf7*) to downy mildew has been identified in *V. vinifera*. In this work, the efficacy of RNAi triggered by exogenous double-stranded RNA (dsRNA) in controlling *P. viticola* infections has been assessed in a highly susceptible grapevine cultivar (Pinot noir) by knocking down *VviLBDIf7* gene. The effects of dsRNA treatment on this target gene were assessed by evaluating gene expression, disease severity, and development of vegetative and reproductive structures of *P. viticola* in the leaf tissues. Furthermore, the effects of dsRNA treatment on off-target (*EF1α*, *GAPDH*, *PEPC*, and *PEPCK*) and jasmonic acid metabolism (*COI1*) genes have been evaluated. Exogenous application of dsRNA led to significant reductions both in *VviLBDIf7* gene expression, 5 days after the treatment, and in the disease severity when artificial inoculation was carried out 7 days after dsRNA treatments. The pathogen showed clear alterations to both vegetative (hyphae and haustoria) and reproductive structures (sporangiophores) that resulted in stunted growth and reduced sporulation. Treatment with dsRNA showed signatures of systemic activity and no deleterious off-target effects. These results demonstrated the potential of RNAi for silencing susceptibility factors in grapevine as a sustainable strategy for pathogen control, underlying the possibility to adopt this promising biotechnological tool in disease management strategies.

## Introduction

Since the late 1800s, *Vitis vinifera* has suffered damage from downy mildew, a disease caused by the oomycete *Plasmopara viticola*, originating from Northern America. This biotrophic, obligate parasite can infect all the green parts of grapevine plants, causing quantitative and qualitative damage and leading to extensive yield losses (Yu et al., 2012). Non-*vinifera* species, such as those with center of origin in North America and Asia, are resistant to *P. viticola* due to coevolution with the pathogen. This resistance is mediated by different mechanisms that at first detect the pathogen and then initiate a proper defense response (Gessler et al., 2011). The first level of defense is called Pathogen-Associated Molecular Pattern (PAMP)-Triggered Immunity (PTI), a plant basal immune response activated by the recognition of conserved molecules of the pathogen (Newman et al., 2013). A second, more selective, plant defense mechanism is called Effector-Triggered Immunity (ETI). ETI relies on a class of highly specific receptors, the resistance proteins (R-proteins), that recognize pathogen effectors. Activation of ETI leads to disease resistance and is often associated with localized apoptosis at the infection site (hypersensitive response) (Jones and Dangl, 2006).

Since effectors and receptors are codified by non-essential genes, resistance genes (R-genes) undergo rapid evolution, due to the strong selective pressure on both pathogen and plant (Buonassisi et al., 2017). This phenomenon implies a short duration of the resistance triggered by R-genes. For a durable resistance, targeting susceptibility genes (S-genes) can be a winning strategy in breeding (Zaidi et al., 2018). S-genes facilitate the compatibility between plant and pathogen and are essential for their interaction, especially for biotrophic pathogens. Therefore, mutation or loss of S-genes can limit pathogenicity toward the plant (Van Schie and Takken, 2014). *MLO* (Mildew Locus O) genes are a striking example of S-gene usefulness in breeding programs: their knockdown confers resistance to powdery mildew in grapevine, reducing the disease severity by up to 77% (Pessina et al., 2016). *MLO-*based resistance to powdery mildew is, indeed, widely employed in barley breeding since few decades even if the function encoded protein is not yet completely established (Kusch and Panstruga, 2017).

To date, downy mildew-resistant grapevine varieties are obtained by crossing *V. vinifera* cultivars with non-*vinifera* species or hybrids. Nevertheless, to reduce the background of non-*vinifera* species, several cycles of backcrossing with susceptible cultivars are needed, which makes the breeding process very lengthy. The identification of grapevine S-genes against *P. viticola* opens new possibilities to breed for downy mildew resistance because usually S-gene-mediated resistance is durable and broad spectrum. The research of S-genes in grapevine is still pioneering. Pessina et al. (2016) identified two *MLO* genes responsible for *V. vinifera* susceptibility to powdery mildew and some others were proposed for downy mildew (Toffolatti et al., 2020; Pirrello et al., 2021). Recently, resistance to *P. viticola* has been identified in Mgaloblishvili (Toffolatti et al., 2016), a *V. vinifera* cultivar from Georgia (Southern Caucasus). Studying its unique resistance mechanism, different R-genes and an interesting candidate S-gene have been identified (Toffolatti et al., 2018, 2020). The S-gene *VvLBDp1* [from here on called *VviLBDIf7* based on Grimplet et al. (2017) nomenclature], encoding for an LOB (LATERAL ORGAN BOUNDARIES) domain-containing (LBD) protein, belongs to plant LOB family of transcription factors. This family has been comprehensively analyzed in many species, such as *Arabidopsis thaliana* (Liu et al., 2005), *Malus domestica* (Wang et al., 2013), *Glycine max* (Yang et al., 2017), *Eucalyptus grandis* (Lu et al., 2018), *Brassica rapa* (Huang et al., 2018), *Camellia sinensis* (Teng et al., 2018), *Gossypium* spp. (Yu et al., 2020), and *Pyrus bretschneideri* (Song et al., 2020). LBD genes show a key role in the regulation of plant organ development and in the response to abiotic and biotic stresses (Xu et al., 2016). They are involved in the establishment of organ boundaries (Rast and Simon, 2012), leaf formation (Semiarti et al., 2001), pulvinus differentiation and petiole development (Chen et al., 2012), regulation of lateral root organogenesis (Okushima et al., 2007), root and stem development (Yu et al., 2020), development of sepal and petal primordia of flowers (Xu et al., 2008), pollen development (Kim et al., 2015), regulation of light/dark-dependent hypocotyl elongation (Mangeon et al., 2011), and secondary phloem growth (Yordanov et al., 2010). Moreover, LOB genes are also involved in pathogen and abiotic response (Thatcher et al., 2012; Grimplet et al., 2017; Yu et al., 2020), and they can act as repressor of anthocyanin biosynthesis and affect nitrogen response (Rubin et al., 2009). Biological processes where LBD genes are involved have been extensively reviewed by Zhang et al. (2020).

A genome-wide characterization has been performed in grapevine as well, and up to 50 LBD genes have been identified (Grimplet et al., 2017). Expression patterns across different tissues, including both mature/woody and vegetative/green tissues, indicate roles of LBD genes in organ differentiation, in berry development and ripening (Fasoli et al., 2012), and in response to abiotic (such as salt, cold and drought) and biotic stresses (such as *Botrytis cinerea* attack and Bois noir disease) (Albertazzi et al., 2009; Agudelo-Romero et al., 2015). The S-gene *VviLBDIf7* is the putative ortholog of an LBD transcriptional factor acting as repressor of jasmonate-mediated defense mechanisms during infection of *A. thaliana* roots with *Fusarium oxysporum*. In this system, LBD disruption resulted in an increased resistance to the pathogen (Thatcher et al., 2012).

Silencing plant S-genes represents a promising way to achieve disease resistance as an alternative or in addition to breeding for R-genes. However, gene silencing is usually obtained *via* stable plant transformation, and the use of genetically modified plants in Europe is strictly regulated, and in several countries, they are not authorized for cultivation. A novel emerging approach, which allows to overcome procedures for a stable genome modification, is represented by RNA interference (RNAi) triggered by the application of exogenous double-stranded RNA (dsRNA) molecules (Dubrovina et al., 2019). The effect of dsRNA treatment has been recently studied and proposed as a new environmentally-friendly crop protection tool from viruses, fungi, and insects (Konakalla et al., 2016; Luo et al., 2017; Wang et al., 2017; Cagliari et al., 2019; Dubrovina and Kiselev, 2019; Morozov et al., 2019; Vadlamudi et al., 2020). RNAi has emerged as a technique with the ability to selectively knock down target genes (Kerschen et al., 2004). RNAi is a natural mechanism used by various organisms, including plants, to regulate specific gene activities or to defend their genome from invasions of exogenous nucleic acids. In the first case, the plant specifically produces molecules called microRNAs (miRNAs) serving as guides to selectively degrade the mRNA of target genes. In the second case, the plant recognizes dsRNAs introduced into the cytoplasm and produces short interfering RNA (siRNA) molecules that defend the plant from exogenous nucleic acids (Meister et al., 2004). As a consequence, RNAi mediated by post-transcriptional gene silencing can be stimulated also by the addition of *ad hoc* designed dsRNA molecules. This technique, although not yet regulated at European level, will likely fall outside the strict GMO regulation, being transgene-free. The exogenous application of polynucleotides that can affect mRNA levels of important virulence-related plants genes without modifying the host genome opens new opportunities for the development of new scientific techniques and crop improvement strategies (Dubrovina et al., 2019). This study evaluated the efficacy of a dsRNA treatment in silencing, through RNAi, *VviLBDIf7*, the candidate S-gene responsible for susceptibility toward *P. viticola* in *V. vinifera*. For this purpose, Pinot noir leaves were treated with synthetized dsRNA targeting *VviLBDIf7* gene and then inoculated with the pathogen, to evaluate (i) *VviLBDIf7* gene expression in the plant tissues after dsRNA treatment, (ii) disease severity and sporangia production on dsRNA-treated leaves inoculated with *P. viticola*, and (iii) the morphology of both vegetative and reproductive *P. viticola* structures in the dsRNA-treated leaf tissues. Finally, a preliminary investigation of the systemic effect of dsRNA and the evaluation of dsRNA treatment on off-target and jasmonic acid metabolism genes have been performed.

## Materials and Methods

### Basal Expression of *VviLBDIf7*

The basal expression level of *VviLBDIf7* (LOC100246173), candidate S-gene, was evaluated on leaves collected from three 6-year-old *V. vinifera* L. cv. Pinot noir plants, grown in a greenhouse, as reported in Toffolatti et al. (2018), *via* RT-qPCR. The first two well-developed leaves were collected in June 2020 twice at 1-day intervals. Leaves were ground with liquid nitrogen into a fine powder using mortar and pestle. RNA was extracted using the Spectrum^TM^ Plant Total RNA Kit (Sigma-Aldrich, Germany) and then digested with Amplification Grade DNase I (Sigma-Aldrich), according to the manufacturer’s instructions. Quantity and quality of RNA were checked by NanoDrop 1000 Spectrophotometer (Thermo Scientific, United Kingdom) and 1% agarose gel electrophoresis stained with Midori Green Advance^®^ (Nippon Genetics, Japan). *VviLBDIf7* real-time PCR primers (VvLBD_F and VvLBD_R; Table 1) were designed on the available sequence [VIT_13s0019g03750 according to Canaguier et al. (2017)] using the Primer3 Plus software (Untergasser et al., 2007) and NCBI Primer-BLAST (Ye et al., 2012). To avoid amplification of eventual genomic DNA carried over, one of the primers was designed to span across the exon–exon junction of the gene. Ubiquitin (Fujita et al., 2007) and actin (Reid et al., 2006) genes were used as references for data normalization. Total RNA (500 ng) was reverse transcribed with SuperScript^®^IV Reverse Transcriptase (Invitrogen, United Kingdom), using a 1:1 mix of random primers and oligo(dT), following manufacturer’s instructions. Real-time PCR reaction was carried out using 4 μl of cDNA diluted 1:10, 10 μl of PowerUp^TM^ SYBR^TM^ Green Master Mix (Applied Biosystems, United Kingdom), 500 nM of primer forward and reverse and water up to 20 μl. Each reaction was performed in triplicate on QuantStudio 3 Real-Time PCR Systems (Thermo Fisher, United Kingdom) using the following cycling conditions: 50°C for 2 min, 95°C for 10 min, 50 cycles at 95°C for 20 s, 60°C for 45 s, and 72°C for 30 s. Each thermal cycle was followed by a melting curve stage, with temperatures ranging from 60°C to 95°C. The *VviLBDIf7* gene expression at the two time points was calculated by comparing 2^–ΔΔ*Ct*^ (Ct = cycle threshold) values (Livak and Schmittgen, 2001). Geometric average of ubiquitin and actin was used to normalize the Ct values.

**TABLE 1:** List of primers used in this work to synthetize the dsRNA.

Name	Sequence (5′-3′)	Tm (°C)	GC %	Primer position (bp)
VvLBD_F	GCCTGCAAAATCCTTCGTCG	60.18	55.0	196–215
VvLBD_R	GACTCGGGAAGTTCCTGCAA	59.97	55.0	322–341
VvLBD_RNAi_F	TATGGTTGTGCTGGTGCAAT	60.00	45.0	406–425
VvLBD_RNAi_R	CACACGGCTCTCCTTTTTCT	59.50	50.0	798–817
VvLBD_RNAiT7_F	TAATACGACTCACTATAGGGAG ATATGGTTGTGCTGGTGCAAT	71.30	41.9	406–425
VvLBD_RNAiT7_R	TAATACGACTCACTATAGGGAG ACACACGGCTCTCCTTTTTCT	72.30	44.2	798–817
VvLBD_2_F	CTTGCAGGAACTTCCCGAGTC	60.94	57.0	321–341
VvLBD_2_R	GCCAAGAAGGCTCCAAGACGG	63.56	62.0	566–586

*VvLBD_F and VvLBD_R primers were used to detect the basal gene expression of VvLBD in leaves of Pinot noir. VvLBD_RNAi_F and VvLBD_RNAi_R primers were used to amplify partially the target gene to synthetize the dsRNA. VvLBD_RNAiT7_F and VvLBD_RNAiT7_R were used to perform MEGAscript RNAi Kit protocol. VvLBD_2_F and VvLBD_2_R primers were used to evaluate the expression of *VvLBD* gene on dsRNA- and water-treated leaves. The primer position refers to the start of the sequence (LOC100246173) annotated in NCBI (https://www.ncbi.nlm.nih.gov/).*

### *VviLBDIf7* dsRNA Design and Synthesis

In order to synthesize the dsRNA molecules, *VviLBDIf7* was partially amplified. To allow the synthesis of dsRNA template of proper dimension (400–500 bp) through PCR, new primers of *VviLBDIf7* (VvLBD_RNAi_F and VvLBD_RNAi_R; Table 1) were designed on the available sequence (LOC100246173) using the Primer3 Plus software and NCBI Primer-BLAST. In order to reduce the probability of off-target amplicons, the partial *VviLBDIf7* sequence was amplified starting from cDNA samples obtained in the *Basal expression of VviLBDIf7* subsection. PCR reaction was carried out using 4 μl of cDNA diluted 1:10, 5 μl of 5X Colorless GoTaq Reaction Buffer (Promega, Wisconsin, United States), PCR Nucleotide Mix 0.2 mM each dNTP (Promega), 0.6 μM of primer forward and reverse, 0.125 μl of GoTaq G2 (5 U/μl) (Promega), and water up to 25 μl. To ensure the production of enough DNA template for the next steps, the reaction was performed in five replicates using the following thermal cycling conditions: 95°C for 2 min, 35 cycles at 95°C for 30 s, 60°C for 1 min, 72°C for 1 min, and a last step at 72°C for 5 min. PCR fragments were visualized on 1% agarose gel electrophoresis stained with Midori Green Advance. The amplification products were used as a template in another PCR reaction using RNAi primers 5′ attached with T7 RNA polymerase promoter sequences (VvLBD_RNAiT7_F and VvLBD_RNAiT7_R; Table 1), as requested by MEGAscript RNAi Kit protocol (Thermo Fisher Scientific, United Kingdom). PCR reaction was carried out using 1 μl of amplification product, 5 μl of 5X Colorless GoTaq Reaction Buffer, PCR Nucleotide Mix 0.2 mM each dNTP, 0.6 μM of primer forward and reverse, 0.125 μl of GoTaq G2 (5 U/μl), and water up to 25 μl. To guarantee a sufficient amount of dsRNA DNA template, the reaction was performed in 50 replicates using the following thermal cycling conditions: 94°C for 5 min, 5 cycles at 94°C for 45 s, 62°C for 1 min, 72°C for 1 min, 30 cycles at 94°C for 45 s, 65°C for 1 min, 72°C for 1 min, and a last step at 72°C for 5 min. PCR products were solved on 1% agarose gel electrophoresis stained with Midori Green Advance. Replicates of PCR product were pooled and purified through Wizard SV Gel and PCR Clean-Up System (Promega). Purified samples were quantified using Qubit^TM^ 3.0 fluorometer (Thermo Fisher Scientific), using the Qubit^TM^ dsDNA HS Assay Kit (Thermo Fisher Scientific), and Sanger sequenced by Macrogen Europe B.V. in two replicates, using RNAiT7 primers. Sequences were aligned to reference sequence using ClustalW (Thompson et al., 1994). The obtained template was used in the dsRNA synthesis reaction using MEGAscript RNAi Kit, according to the manufacturer’s instructions. Synthetized dsRNA was solved on 1% agarose gel electrophoresis stained with Midori Green Advance and quantified by NanoDrop 1000 Spectrophotometer.

### *VviLBDIf7* dsRNA Treatment

**FIGURE 1:**
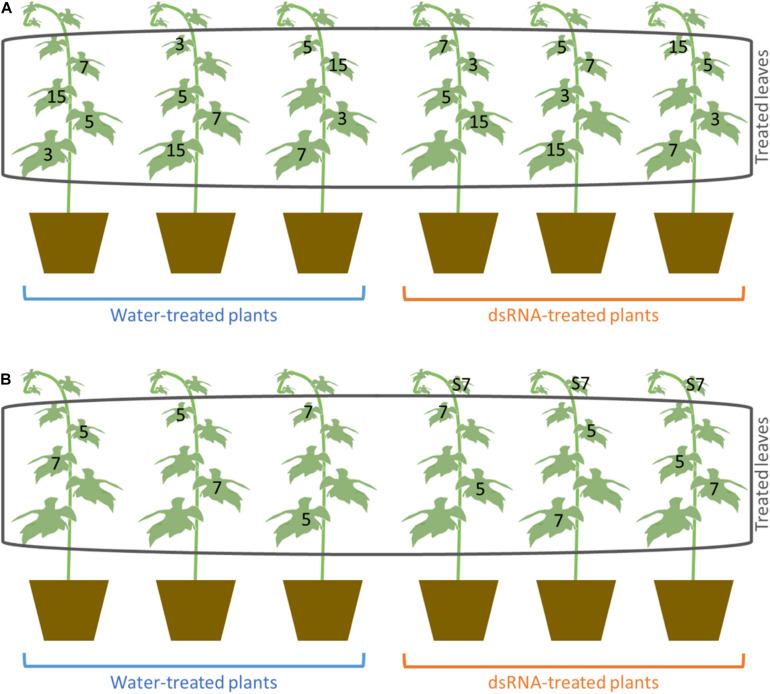
Scheme of the treatment and sampling carried out in the first **(A)** and second experiment **(B)**. Numbers indicate the number of days after treatments (dat) at which the leaves were collected. S7 indicates the untreated leaves that were sampled at 7 dat for the evaluation of systemic activity of dsRNA in the second experiment.

Treatments with *VviLBDIf7* dsRNA were carried out on 6-year-old Pinot noir plants grafted onto SO4, maintained in a glasshouse in 5-L pots filled with sand-peat mixture (7:3v/v), regularly watered *via* a drip system and fertilized twice a year with Osmocote Topdress fertilizer (ICL Specialty Fertilizers, Italy), as reported in Toffolatti et al. (2018). The plants, grown in a greenhouse, never came in contact with *P. viticola* structures and were regularly inspected for disease symptoms to be sure to work with healthy tissues. Three plants were treated with 100 μg/plant of dsRNA dissolved in 1 ml of sterilized water, and three were treated with only sterilized water (1 ml per plant), as suggested by Nerva et al. (2020). Treatments were performed with an airbrush on both sides of the first five fully developed leaves of a single vine shoot (from the second to the sixth leaf from the apex of the shoot) per plant (Supplementary Figure 1). One sprayed leaf per plant was randomly collected at 3, 5, 7, and 15 days after treatment (dat) (Figure 1A). To validate the dsRNA treatment data, the experiment was repeated, as previously described, on self-rooted cuttings of Pinot noir grown in the same conditions, to remove any possible effect of rootstock on scion behavior. In this second experiment, leaves were collected at 5 and 7 dat (Figure 1B), corresponding to the time points when, according to the first experiment, downregulation of S-gene and response to the pathogen occurred. In the second experiment, the possible systemic effect of dsRNA treatment was investigated on the untreated leaf immediately above the treated one at 7 dat (leaf S7, Figure 1B). From each leaf (biological replication), three 1.5-cm-diameter disks (technical replication) were excised and inoculated with the pathogen. The remaining leaf tissue was frozen in liquid nitrogen and stored at −80°C until gene expression analysis and assessment of *P. viticola* presence.

### *VviLBDIf7* Gene Expression Analysis on dsRNA- and Water-Treated Leaves

Expression of the candidate S-gene on dsRNA- and water-treated samples was evaluated through RT-qPCR. RNA extraction, RT-qPCR, and comparison of Ct values were carried out following the procedures described in the *Basal expression of VviLBDIf7* subsection, with some modifications: (i) to ensure the specific retrotranscription of only functional mRNAs, oligo(dT) primers were used in RT-PCR; (ii) qPCR was performed on 8 μl of cDNA; and (iii) to avoid possible amplification of incomplete cDNA sequences due to amplification of small RNAs resulting from degradation of the target gene mRNA following dsRNA treatment, qPCR primers (VvLBD_2_F and VvLBD_2_R; Table 1) were specifically designed, using Primer3 Plus software and NCBI Primer-BLAST. The forward primer was designed to match the target gene sequence in the middle of the region amplified by the first qPCR primer pair (see the *Basal expression of VviLBDIf7* subsection), and the reverse primer was designed to match the same sequence region targeted by the dsRNA fragment.

### Inoculation of *Plasmopara viticola* on Treated Leaves and Phenotypic Characterization of the Plant–Pathogen Interaction

Experimental inoculation of the leaf disks with the pathogen were carried out by mixing *P. viticola* sporangia coming from Western (S. Maria della Versa, Pavia) and Eastern (Casarsa della Delizia, Pordenone) Italian field populations (Maddalena et al., 2020; Sargolzaei et al., 2020). To verify that the leaf tissues used for the experimental activities were not previously contaminated by *P. viticola*, a PCR assay with primers specific for the ITS region of *P. viticola* (Toffolatti et al., 2012) was carried out on all leaf samples. Leaf tissues were collected from each sample (dsRNA- and water-treated) at all-time points. DNA was extracted using the DNeasy Plant Mini kit (Qiagen Italia, Milano) and checked by NanoDrop for quantity and quality. PCR reactions were performed using 2 μl of DNA template, 12.5 μl of DreamTaq Green PCR Master Mix (2X) (Thermo Fisher Scientific), 0.5 μM of forward (AF_2F: 5′-TCCTGCAATTCGCATTACGT-3′) and reverse (AF_2R: 5′-GGTTGCAGCTAATGGATTCCTA-3′) primers (Toffolatti et al., 2012), and water up to 25 μl. The thermal cycling conditions were as follows: 94°C for 3 min, 30 cycles at 94°C for 30 s, 57°C for 30 s, 72°C for 1 min, and a last step at 72°C for 5 min. PCR products were solved on 2.5% agarose gel electrophoresis and stained with Realsafe nucleic acid staining solution (Real, Valencia). Positive control consisted of DNA extracted from sporangia and infected leaf disks and negative controls consisted of water and *B. cinerea* DNA.

Leaf disks, sampled at each time point, were placed, lower surface upward, in a Petri dish (9 cm diameter) containing moistened filter paper. The leaf disks were airbrushed with 0.2 ml of a sporangia suspension (5 × 10^4^ sporangia ml^–1^) obtained by collecting sporangia in sterile distilled water, and incubated in a growth chamber at 22°C with a 12-h photoperiod (Toffolatti et al., 2018). The effect of dsRNA treatment on the pathogen’s ability to infect the leaf tissues was evaluated at 7 days after inoculation by combining quantitative (disease severity and the production of sporangia) and qualitative traits, related to the morphology of vegetative and reproductive structures of the pathogen. Disease severity was evaluated in both experiments, while sporangia production and microscopy were performed in the first experiment only. The disease severity of each biological replicate was estimated from the percentage of leaf disk area covered by sporulation (PSA) (Toffolatti et al., 2012). The number of sporangia produced by the pathogen per leaf unit (sporangia cm^–2^) was determined as described by Toffolatti et al. (2016) by collecting the sporangia from each leaf disk in 1 ml of 20% glycerol:water (v:v) and counting them in Kova chambers. Microscopy observations were performed by aniline blue staining (Wick, 2009) on leaf disks fixed in absolute ethanol and cleared as described by Alexander et al. (2005) with some modifications: samples were boiled in 85% ethanol:water (v:v) for 10 min, and incubated in pre-warmed lactic acid at 70°C for 30 min, following the procedure reported by Ricciardi et al. (2021). Reagents were purchased from Sigma-Aldrich. Samples were observed under an EasyLab CX40 (Olympus) bright-field optical microscope equipped with Primo Cam HD5 camera (Tiesselab, Milano, Italy). Pictures were taken as Z sections and overlapped by using ImageJ software^1^.

### Evaluation of dsRNA Treatment on Off-Target and Jasmonic Acid Metabolism Genes

In order to evaluate the effect of dsRNA treatment on off-target genes, the following genes were evaluated *via* RT-qPCR: *EF1α* (elongation factor 1α; EF1α_F: 5′-GAACTGGGTGCTTGATAGGC-3′; EF1α_R: 5′-ACCAAAATA TCCGGAGTAAAAGA-3′) and *GAPHD* (glyceraldehyde-3-phosphate dehydrogenase; GAPHD_F: 5′-TCAAGGTCAAGGA CTCTAACACC-3′; GAPHD_R: 5′-CCAACAACGAACATA GGAGCA-3′) (Figueiredo et al., 2015); *PEPC* (phosphoenolpyruvate carboxylases; PEPC_F: 5′-CATGAAGG GTATTGCTGCTG-3′; PEPC_R: 5′-AGAGGATTTGA TTTTGGTACGG-3′) and *PEPCK* (PEP carboxykinases; PEPCK_F: 5′-TGGCTGGTCAACACTGGTTG-3′; PEPCK_R: 5′-CTTCAGAAGGCTTCCAGAGTG) (Sweetman et al., 2012). Gene expression analysis was performed on dsRNA- and water-treated leaves at 3, 5, 7, and 15 dat (first experiment). On the same samples, the *COI1* (coronatine insensitive 1; COI1_F: 5′-ATGCCCATAGTATTCCCTTTT; COI1_R: 5′-GAACTTCTAATCCTCTGTCTC-3′) and *JAR1* (jasmonate-resistant 1; JAR1_F: 5′- GAGAATTGCGGATGGTGATA-3; JAR1_R: 5′-CTAAAGGCGAAAGAGGTT-3′) (Figueiredo et al., 2015) genes, involved in the jasmonic acid metabolism and modulated by *P. viticola* infection, were investigated *via* RT-qPCR. For more details about the reaction conditions, please refer to the *Basal expression of VviLBDIf7* subsection. Annealing temperature was set to 58°C.

### Statistical Analysis

The 2^–ΔΔ*Ct*^ values were subjected to Levene’s test to assess homogeneity of variance in R software. LSD (least significant difference) test was performed in R to evaluate (i) *VviLBDIf7* basal expression in leaves, (ii) differences among dsRNA- and water-treated leaves in *VviLBDIf7* gene expression values, and (iii) differences among dsRNA- and water-treated leaves in expression values of off-target and jasmonic acid metabolism genes.

ANOVA was carried out with IBM SPSS v.25 software on (i) transformed PSA values $[\text{asin}\left(\frac{\sqrt{P\,S\,A}}{100}\right)$] to establish the existence of significant differences among dsRNA- and water-treated samples at each sampling time, and (ii) sporangia cm^–2^ values to establish the existence of significant differences between dsRNA- and water-treated samples at each dat.

All the results were plotted in bar plots generated by SPSS v.25 software.

## Results

### dsRNA Treatment Decreases *VviLBDIf7* Gene Expression

**FIGURE 2:**
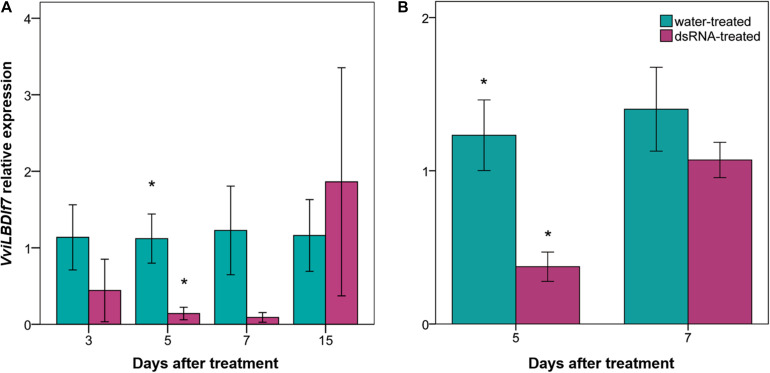
Effect of dsRNA treatment on *VviLBDIf7* gene expression. *VviLBDIf7* gene expression analysis determined based on the 2^–ΔΔ*Ct*^ method at 3, 5, 7, and 15 days after treatment (dat) on Pinot noir plants grafted onto SO4 **(A)**, and at 5 and 7 dat on self-rooted Pinot noir plants **(B)**. Bars represent standard errors. Asterisks indicate statistically significant differences among the dsRNA- and water-treated conditions at each time point (*p value = 0.05).

To assess the basal expression level of *VviLBDIf7* gene, RT-qPCR was performed on Pinot noir leaves collected at 1-day intervals. Results highlighted that the *VviLBDIf7* gene is constitutively expressed in leaves of Pinot noir plants grown in the glasshouse: there was no significant variation in basal expression level in the examined time points (0.45 ± 0.11 *versus* 0.83 ± 0.23, for first and second sampling, respectively, *p* value > 0.05), suggesting a relatively constant expression of the candidate S-gene. A dsRNA 412-bp long has been synthesized on the *VviLBDIf7* gene sequence of Pinot noir to be applied in the RNAi experiment (Supplementary file 1). Pinot noir plants grafted onto SO4 were treated with the dsRNA and the knocking down of the target gene was assessed at 3, 5, 7, and 15 days after dsRNA treatment (dat). A decrease in *VviLBDIf7* expression, even if not statistically significant, was observed between dsRNA- and water-treated leaves at 3 dat, and a statistically significant expression reduction was observed at 5 dat (Figure 2A). At 7 and 15 dat, no significant differences were detected between samples, indicating a progressive end of the transient dsRNA effect (Figure 2A). The experiment was repeated treating self-rooted cuttings of Pinot noir and collecting leaves at 5 and 7 dat. Statistically significant differences in the expression levels of *VviLBDIf7* gene were observed between dsRNA- and water-treated leaves at 5 dat also in the second trial (Figure 2B). No significant difference was observed at 7 dat (Figure 2B).

The dsRNA treatment induced no visible negative effects on grapevine plants, which kept their normal phenotypic traits and vigor.

### dsRNA Treatment Reduces Pathogen Infection and Sporulation

The complete absence of pathogen DNA was observed in the analysis of the leaves used for the experimental inoculations with *P. viticola*, confirming that the samples were healthy before inoculation. An example of the results obtained is reported in Supplementary Figure 2. To investigate whether the dsRNA treatment induced changes in the downy mildew disease extent, dsRNA- and water-treated leaf tissues were experimentally inoculated with *P. viticola* to evaluate phenotypic traits such as disease severity, estimated through PSA, sporangia production, and morphology of pathogen structures. In the first experiment, phenotyping was carried out at 3, 5, 7, and 15 dat; in the second, at 5 and 7 dat.

**FIGURE 3:**
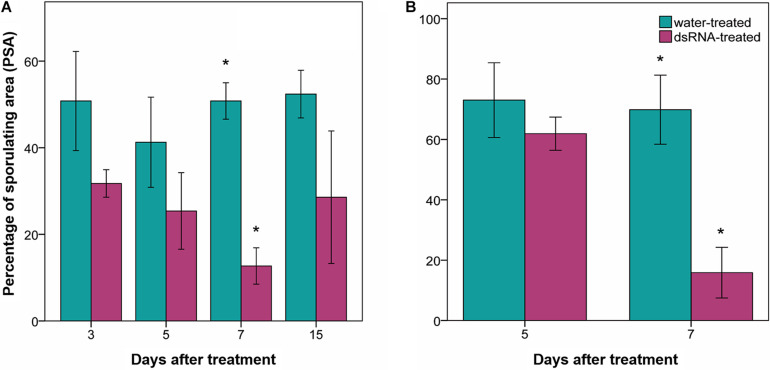
Effect of dsRNA treatment on percentage of sporulating area (PSA) evaluated 7 days after *Plasmopara viticola* inoculation. PSA evaluated on Pinot noir plants grafted onto SO4 at 3, 5, 7, and 15 days after treatment (dat) **(A)**, and on self-rooted Pinot noir plants at 5 and 7 dat **(B)**. Bars represent standard error. Asterisks indicate statistically significant differences among the dsRNA- and water-treated conditions at each time point (*p value < 0.036).

Percentage of leaf disk area covered by sporulation values of water-treated samples ranged from 41 to 52% (Figure 3A) and from 70 to 73% (Figure 3B) in the first and second experiment, respectively. No significant differences (0.8 < *F* < 2.5; df = 1–4; *p* > 0.22) were found among PSA values of dsRNA- and water-treated samples inoculated at 3, 5, and 15 dat (Figure 3A). Instead, the PSA values of the water-treated leaves inoculated at 7 dat were four times higher, with statistical significance (9.7 < *F* < 29.6; df = 1–4; *p* < 0.036), than those recorded on dsRNA-treated samples inoculated at 7 dat in both experiments (Figures 3A,B).

**FIGURE 4:**
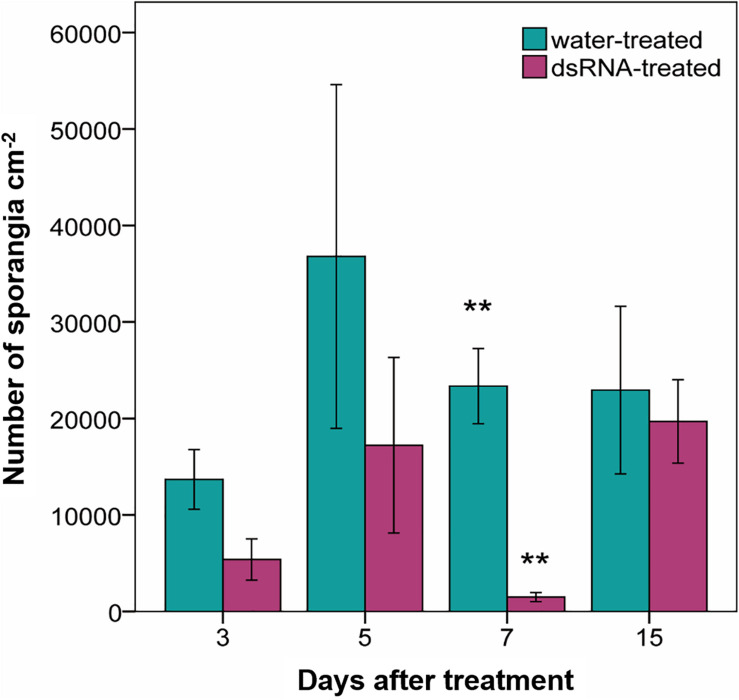
Production of sporangia (number of sporangia cm^–2^) evaluated 7 days after *Plasmopara viticola* inoculation on dsRNA- and water-treated leaves at 3, 5, 7, and 15 days after treatment of Pinot noir plants grafted onto SO4. Bars represent standard error. Asterisks indicate statistically significant differences among the dsRNA- and water-treated conditions at each time point (**p value 0.005).

Sporangia production in the water-treated leaf disks ranged from 13,683 ± 2959 (average ± standard error) to 36,801 ± 17,849 sporangia cm^–2^ and did not significantly differ from those of the dsRNA-treated samples at any time from treatment (*F* < 4.9; df = 1–4; *p* > 0.09) apart from 7 dat (*F* = 31; df = 1–4; *p* = 0.005), when sporangia production was significantly reduced to 1,486 ± 316 sporangia cm^–2^ in the dsRNA-treated samples (Figure 4).

**FIGURE 5:**
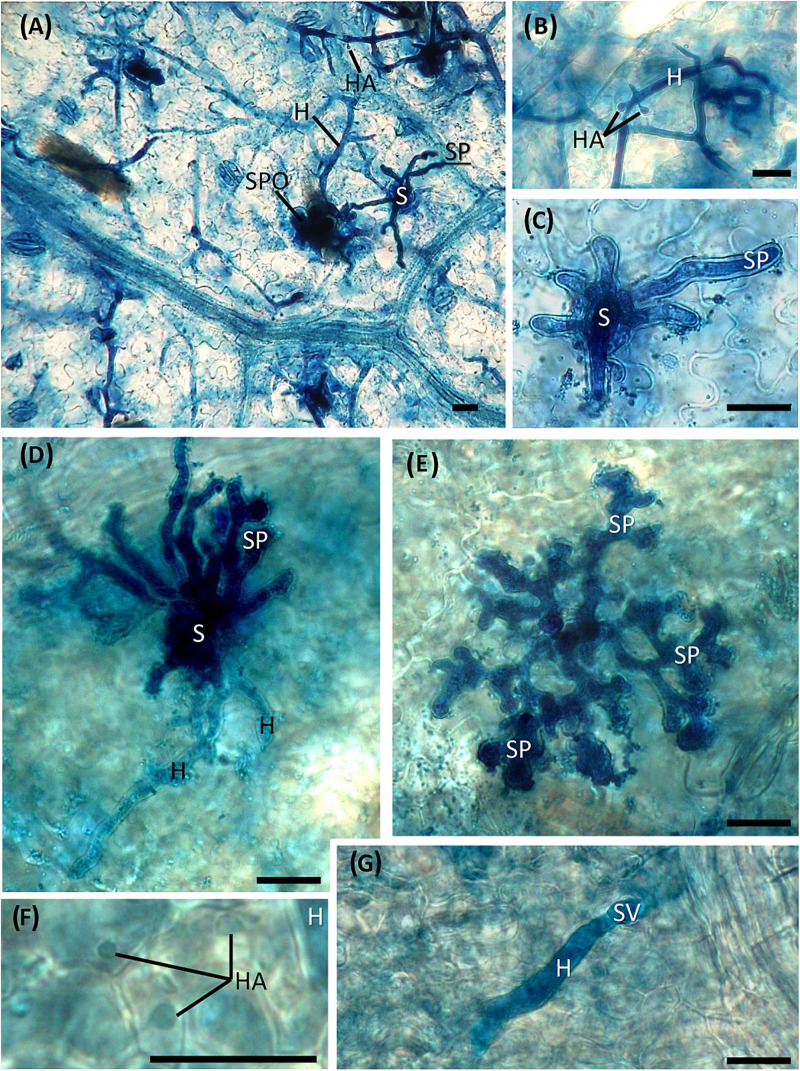
Development of *Plasmopara viticola* structures inside untreated **(A–C)** and treated **(D–G)** leaf tissues inoculated at 7 dat. **(A)** Hyphae with haustoria developing in the mesophyll cells and sporangiophores emerging from the stomata. **(B)** Mycelium with haustoria; **(C)** detail of sporangiophores emerging from a stoma; **(D)** degenerating hyphae, slightly colored and vacuolized, with no visible haustoria, and hyperbranched sporangiophores emerging from the stoma; **(E)** short, hyperbranched and sterile sporangiophores; **(F)** light-colored haustoria; **(G)** hypha developing from the substomatal vesicle with no visible haustoria. S = stoma; H = hypha; HA = haustorium; SP = sporangiophore; SPO = sporangium; SV = substomatal vesicle. Scale bar, 20 μm.

The development of vegetative and reproductive structures of *P. viticola* in the leaf tissues was observed at the microscope by using a staining procedure with aniline blue dye, which allowed us to observe the pathogen structures in bright field. Hyphae with numerous haustoria, also clearly visible at low magnification (Figures 5A,B), regularly developed in the intercellular spaces of the lacunose space of the water-treated leaf tissues (Figures 5A–C). Sporangiophores emerged from the stomata (Figure 5C) and numerous sporangia could be observed on the leaf surface (Figure 5A). Alterations in the pathogen structures were clearly visible in the dsRNA-treated leaves only at 7 dat (Figures 5D–G): hyphae had a reduced diffusion (Figure 5G) and appeared lightly colored and highly vacuolated (Figure 5D); haustoria were lightly colored and visible only at high magnification (Figure 5F); short, hyperbranched, and sterile sporangiophores emerged from the stomata (Figure 5E). The same alterations were seen in all the points where the pathogen attempted penetration, indicating a uniform response of the leaf tissues to dsRNA treatment and pathogen inoculation.

The PSA values of the untreated leaves sampled immediately above the dsRNA-treated leaves (leaf sample S7; Figure 1B) at 7 dat in the second experiment (PSA = 21.4% ± 7.1 SD) did not significantly differ (*F* = 5.3; df = 1–4; *P* = 0.08) from the PSA values recorded in the dsRNA-treated leaves (PSA = 14.6% ± 8.4 SD) and significantly differed (*F* = 15.8; df = 1–4; *P* = 0.016) from the PSA values of the water-treated leaves (PSA = 69.8% ± 11.4 SD) sampled at the same time point.

### Treatment With dsRNA Does Not Reduce Expression of Off-Target Genes

**FIGURE 6:**
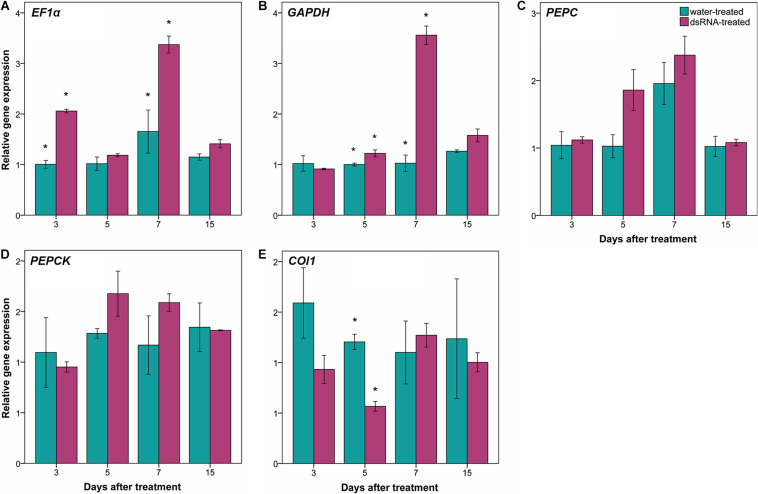
Effect of dsRNA treatment on *EF1α* [elongation factor 1α; **(A)**], *GAPHD* (glyceraldehyde-3-phosphate dehydrogenase; B), *PEPC* [phosphoenolpyruvate carboxylases; **(C)**], *PEPCK* [PEP carboxykinases; **(D)**], and *COI1* [coronatine insensitive 1; **(E)**] gene expression at 3, 5, 7, and 15 days after treatment (dat) on Pinot noir plants grafted onto SO4. Gene expression analysis determined based on the 2^–ΔΔ*Ct*^ method. Bars represent standard errors. Asterisks indicate statistically significant differences among the dsRNA- and water-treated conditions at each time point (*p value = 0.05).

The effect of dsRNA treatment has been evaluated in the gene expression of some off-target genes: *EF1α*, *GAPHD*, *PEPC*, and *PEPCK* (Figure 6). Relative gene expression of *PEPC* and *PEPCK* genes did not show statistical significant differences between dsRNA- and water-treated leaves at any time points (Figures 6C,D). Statistical significant differences between dsRNA- and water-treated leaves have been detected at 3 and 7 dat for *EF1α* gene (Figure 6A) and at 5 and 7 dat for *GAPHD* gene (Figure 6B), with expression values of dsRNA-treated leaves higher than the water-treated ones.

Regarding the effect of dsRNA treatment on jasmonic acid metabolism genes, the relative gene expression of two genes (*COI1* and *JAR1*) has been investigated. *JAR1* primers did not amplify any fragments at our amplification conditions (annealing temperature = 58°C), while aspecific fragments have been amplified at lower annealing temperature (data not shown). Amplification of *COI1* gene showed statistically significant differences between dsRNA- and water-treated leaves only at 5 dat, with expression values of dsRNA-treated leaves lower than the water-treated ones (Figure 6E).

## Discussion

### Foliar-Applied dsRNA Reduces Expression of *VviLBDIf7* Gene

All plant genes involved in facilitating pathogen infection and supporting a compatible plant–pathogen interaction are considered S-genes (Van Schie and Takken, 2014); therefore, silenced S-genes no longer support a compatible plant–pathogen interaction and can cause pathogen-specific resistance (Pavan et al., 2010; Thatcher et al., 2012).

RNAi is a post-transcriptional gene silencing mechanism triggered by dsRNA molecules to prevent the expression of target genes (Kim and Rossi, 2007). The exogenous application of dsRNAs targeting essential interaction genes in plants (S-genes) or plant pathogens and pests has been successfully used to both control diseases and induce gene silencing as a valid alternative to genetic transformation (Mitter et al., 2017; Ghosh et al., 2018; Rosa et al., 2018; Dubrovina and Kiselev, 2019; Gu et al., 2019; Dalakouras et al., 2020). In this work, the efficacy of RNAi approach in knocking down the putative grapevine S-gene (*VviLBDIf7*) identified by Toffolatti et al. (2020) in grapevine–*P. viticola* interaction has been investigated. A prerequisite for setting up a successful foliar RNAi experiment to knock down the target gene is the expression of the target gene in leaves at any stages, regardless of environmental conditions. Therefore, the first step of this study was the evaluation of S-gene basal expression. RT-qPCR data showed that the expression of *VviLBDIf7* in Pinot noir leaves did not change significantly over time in the absence of perturbing conditions, confirming that S-genes can show a constitutive expression (Eichmann et al., 2004).

Foliar-applied dsRNA molecules targeting plant *VviLBDIf7* gene proved to knock down *VviLBDIf7* gene expression in Pinot noir, a grapevine cultivar susceptible to *P. viticola* infection, at 5 dat. The RNAi triggered by exogenous dsRNA is known to be transient, lasting from a few days up to a couple of weeks (Dalakouras et al., 2016, 2020; Niehl et al., 2018; Dubrovina and Kiselev, 2019; Nerva et al., 2020). In this interval, the efficiency peak of the dsRNA treatment is affected by several factors, determining the absorption rate of the exogenous molecules by plant cells, including dsRNA concentration, dose and length, application method, delivery technique, plant organ-specific activities, and stability of the molecule under unfitting environmental conditions (Das and Sherif, 2020). In our experimental conditions, evaluations performed at 7 (both experiments) and 15 (first experiment) dat indicated a progressive reduction of the transient dsRNA effect in knocking down the expression of *VviLBDIf7* gene. Instead, knockdown at 5 dat has been confirmed by the second experiment as well, when self-rooted plants were used. This second approach was used to validate the results, avoiding any rootstock interference. Rootstock can affect the scion behavior at different levels, such as plant development, biomass accumulation and phenology (Ollat et al., 2015). These results demonstrated that the transient effect of dsRNA was not affected by rootstock and that in our conditions, the RNAi is terminated by 15 dat.

Fifty LBD genes have been identified in grapevine genome (Grimplet et al., 2017), expressed in different tissues (such as young leaves, developed tendril, and inflorescences), in berry development and ripening and in response to abiotic and biotic stresses (Albertazzi et al., 2009; Fasoli et al., 2012; Agudelo-Romero et al., 2015). *VviLBDIf7* seems to be expressed at low levels in all mature/woody and vegetative/green tissues, while it appears to be up-regulated in berries upon *B. cinerea* attack (Agudelo-Romero et al., 2015). In our experimental conditions, no negative effects could be observed in grapevine plants treated with dsRNA: this could be due to the transient effect of RNAi. The presence of pleiotropic effects on biological processes and in response to abiotic and biotic stresses (not yet investigated) associated with *VviLBDIf7* silencing should be better investigated in future studies on stable grapevine transformants.

### Reduction of *VviLBDIf7* Expression Is Followed by Reduced Susceptibility to *Plasmopara viticola*

The developed staining protocol has two main advantages: firstly, the staining is stable and does not fade away rapidly, as normally occurs when using aniline blue staining techniques for fluorescence microscopy (Díez-Navajas et al., 2007), allowing the operator to perform a more thorough investigation of the pathogen structures; secondly, the observations can be performed with a microscope that is normally present in a basic mycology laboratory, without the need for a fluorescence microscope. Aniline blue binds to β-glucans (β-1,3-glucans in particular) located in the *P. viticola* cell wall, staining the pathogen structures in blue. However, also plant cell walls can be stained, especially callose depositions (Hood and Shew, 1996) that are frequently found as a defense reaction to the invading pathogen (Trouvelot et al., 2008), causing some faint blue staining also in plant tissues. No differences could be observed between treatments in pathogen growth and sporulation at all time point except at 7 dat (2 days after downregulation of *VviLBDIf7*), when Pinot noir leaves treated with dsRNA showed a significant reduction in disease severity as well as impairment in *P. viticola* growth and sporulation, compared to the water-treated leaves. In the dsRNA-treated samples, the alterations in the vegetative structures of the pathogen were evident. The hyphae did not freely diffuse inside the leaf tissues but developed only in the area immediately surrounding the infection point and led to the differentiation of hyperbranched, partly sterile, sporangiophores. Analogous alterations were observed as a consequence of resistance response in Mgaloblishvili (Toffolatti et al., 2018) and in response to environmental (Rumbolz et al., 2002) and chemical (Ricciardi et al., 2021) stresses. The vacuolation of the hyphae underneath the sporangiophore could be associated with the necessity of the mycelium to provide material to support sporulation, as occurs in true fungi (Thrane et al., 2004), or to a degradation of the mycelium. Abnormal hyphal vacuolation has been observed in hyphae of the oomycete *Saprolegnia ferax* following the application of growth inhibitors (Bachewich and Heath, 1999). Moreover, the vegetative structures of the pathogen in the dsRNA-treated samples appeared more lightly colored compared to the water-treated samples. Since aniline blue binds to β-glucans, this discoloration could indicate an alteration in the cell wall composition. While this altered mycelium was clearly visible following aniline blue staining, haustoria were less colored and hardly recognizable. This could indicate that the dsRNA treatment primarily affected the haustoria, which are the only structures of the pathogen that actively interact with the host cell (Toffolatti et al., 2011). *P. viticola* is an obligate parasite of grapevine and does not directly damage the host cell, the activity of which must be preserved to allow the absorption of nutrients. Alterations at haustoria level can induce a reduced pathogen growth not only because they have a role in nutrient uptake, but also because they represent a specialized interface for delivering effectors to plants that are associated with resistance (Judelson and Ah-Fong, 2019). More accurate investigation on the haustoria ultrastructure is needed to confirm these preliminary results. The alteration in the differentiation of sporangiophores was associated with a significant reduction in the sporangia production.

The analogous disease severity of the pathogen observed in the second experiment both on the leaves that were directly treated with dsRNA and those directly above the treated leaves (systemic diffusion), which was significantly lower than that achieved in the water-treated samples, indicates the presence of a systemic effect of the treatment that should be more deeply investigated in future studies. Systemic RNAi has been observed in other plants, such as *A. thaliana* (Melnyk et al., 2011), *Hordeum vulgare* (Biedenkopf et al., 2020), and *Nicotiana benthamiana* (Chen et al., 2018), but to the best of our knowledge, no information is reported for grapevine.

### *VviLBDIf7* Is a Candidate Gene to Be Silenced to Reduce Downy Mildew Susceptibility

A potential limitation of RNAi approach can be the possible effect on off-target genes. Off-target effects occur when a siRNA down-regulates unintended targets. For this reason, it is useful to assess off-target potential in order to avoid undesirable phenotypes (Rosa et al., 2018). In this work, dsRNA treatment did not reduce the expression of *EF1α*, *GAPHD*, *PEPC*, and *PEPCK* off-target genes. The up-regulation of *EF1α* and *GAPHD* genes in dsRNA-treated leaves at some time points can be a consequence of the knockdown of the *VviLBDIf7* gene (the plant reacts to the gene silencing by improving its basal metabolism).

In *A. thaliana*, the *lbd20* mutant showed a reduced disease severity after *F. oxysporum* infection and a modulation of jasmonic acid-mediated defense genes (Thatcher et al., 2012). In grapevine, some evidences of jasmonic acid involvement in resistant cultivar in response to *P. viticola* infection have been described (Figueiredo et al., 2015; Li et al., 2015). After *P. viticola* inoculation, Figueiredo et al. (2015) observed an up-regulation of jasmonic acid biosynthesis-related genes (*LOXO*, *AOS*, *AOC*, and *OPR3*) at 6 and 12 hpi (hours post-infection), and an up-regulation of genes involved in the jasmonic acid activation and signaling (*JAR1* and *COI1*, respectively) at 18 and 24 hpi. In this work, due to the sampling times (3, 5, 7, and 15 dat), only the gene expression profile of *JAR1* and *COI1* genes has been investigated. Although we have to refer to only *COI1* gene expression data (no amplification fragments were detected for *JAR1* gene), the jasmonic acid pathway appears to be down-regulated, as *COI1* gene expression values at 5 dat in dsRNA-treated leaves were lower than in the water-treated ones. These data confirm what has been observed for *LBD20* gene in *A. thaliana*, where *coi1* mutants did not induce *LBD20* gene expression, and support the role of LBD genes in jasmonic acid signaling (Thatcher et al., 2012).

## Conclusion

The results reported in this work highlight the great potential of RNAi-based strategies in sustainable defense management. In the current scenario, the treatment with fungicides still represents the most effective agronomical practice to defend vineyards by *P. viticola* attack. Nevertheless, Directive 2009/128/EC and Regulation (EC) No. 1107/2009 of the European Parliament and of the Council concerning the placing and use of plant protection products on the market impose to the farmers the reduction of fungicide applications, due to their negative impact on human health and environment. In this view, the development of novel and sustainable disease management strategies is essential. Numerous efforts are ongoing with the aim to obtain resistant varieties, exploiting alternative strategies to classical biotechnological tools, such as GMOs (genetically modified organisms), which are currently subjected to strict regulation (Capriotti et al., 2020). For this purpose, the use of RNAi for silencing plant susceptibility genes, which facilitate infection and support compatibility (Van Schie and Takken, 2014), represents a promising alternative to traditional means, such as fungicides, for disease control. Indeed, the phenotypic characterization of quantitative and qualitative *P. viticola* traits allowed us to establish the efficacy of exogenous dsRNA application in silencing the *VviLBDIf7* gene, which led to a reduced pathogen growth and sporulation rate in Pinot noir, a cultivar that is normally highly susceptible to the pathogen. Based on gene expression and PSA values, the RNAi effect is concluded by 15 dat indicating that further treatments are needed for the subsequent disease control. The signatures of systemic activity shown in the present study suggest that the dsRNA treatment could also reach untreated parts of the plant, a feature that is highly desirable since grapevine has an important growth in open field and systemic properties of RNAi could allow the protection of the newly formed vegetation not covered by the treatment. Further investigation is, however needed to clarify the movement inside the plant as well as the duration and the efficacy of dsRNA on untreated parts of the plant. Moreover, considering the high heterozygosity and the varietal rigidity imposed by registered designations of origin that affect development and cultivation of resistant varieties obtained by crossing non-*vinifera* species with *V. vinifera*, the use of RNAi approach in grapevine could represent a valid tool for specifically targeting a known gene. Further implementation of the method is, however, needed to improve the delivery of the dsRNA and achieve a more rapid silencing of the gene that could be compatible with a field use, although an appropriated regulation of topical RNAi applications is still missing (Mezzetti et al., 2020). For an effective field application of dsRNAs as a disease management tool, the following aspects need to be optimized: (i) the concentration and length of dsRNA molecules, (ii) the formulation that should prevent dsRNA degradation (encapsulation could solve this issue) and allow the uptake of dsRNAs into cells, (iii) the delivery strategy (high-pressure spraying or brush-mediated leaf applications), and (iv) the efficacy of dsRNA recognition by the RNAi pathway of the target organism. Furthermore, this approach that uses dsRNA to silence the susceptibility gene of the plant instead of targeting an essential gene of the pathogen should reduce the possible cross-species effect of the dsRNA (e.g., on beneficial microorganisms) and the development of resistances in the pathogen population. Overall, RNAi-mediated silencing could make a great contribution toward integrated pest management, which, taking a holistic approach that exploits all the available disease management tools (i.e., resistant varieties, agronomic practices, and chemical and non-chemical pathogen control), represents the most effective way to manage diseases.

## Data Availability Statement

The raw data supporting the conclusions of this article will be made available by the authors, without undue reservation.

## Author Contributions

GDL and SLT conceived the study. GDL, SLT, AP, and PC set up the experiment. VR, AP, and GDL performed molecular biology analysis. SLT, DM, and EMF performed the phenotypic characterization of *P. viticola.* GDL, SLT, and GM wrote the manuscript. AP, PC, PAB, and OF critically revised the manuscript. All the authors reviewed and approved the manuscript before submission.

## Conflict of Interest

The authors declare that the research was conducted in the absence of any commercial or financial relationships that could be construed as a potential conflict of interest.

## Footnotes

1 https://imagej.nih.gov/ij/index.html
