# Supplementary material for: RNAi of a Putative Grapevine Susceptibility Gene as a Possible Downy Mildew Control Strategy
Source: Front Plant Sci. 2021 May 28;12:667319. doi: 10.3389/fpls.2021.667319 (PMC8196239; doi:10.3389/fpls.2021.667319)
Supplement: Supplementary file 1 [file Data_Sheet_1.DOCX]

***Supplementary materials***

**Supplementary file 1.** dsRNA sequence, 412 bp long, targeting *VviLBDIf7* S-gene.

**Supplementary Figure 1.** Scheme of the leaf choice and treatment. A) Leaves chosen for the water/dsRNA treatment (2^nd^ to the 6^th^ fully developed leaf from the apex of the shoot). B) Spraying of the water/dsRNA treatment on the upper side of the leaf. C) Spraying of the water/dsRNA treatment on the underside of the leaf. For each treatment, a single leaf per plant was randomly sampled at different time points (3, 5, 7 and 15 dat in the first experiment; 5 and 7 dat in the second experiment) for the experimental inoculation with *P. viticola*.

**Supplementary Figure 2.** Picture of the agarose gel showing the absence of amplification in some of the leaf samples used for the experimental inoculation of *P. viticola* and in negative controls (*Botrytis cinerea* DNA and water) and the presence of the band corresponding to *P. viticola* samples (sporangia and infected leaf). Ladder 50 bp biotechrabbit GmbH, Henningsdorf DE.
