# Supplementary material for: RNAi of a Putative Grapevine Susceptibility Gene as a Possible Downy Mildew Control Strategy
Source: Front Plant Sci. 2021 May 28;12:667319. doi: 10.3389/fpls.2021.667319 (PMC8196239; doi:10.3389/fpls.2021.667319)
Supplement: Supplementary file 2 [file Data_Sheet_2.DOCX]

**Supplementary file 1.** dsRNA sequence, 412 bp long, targeting *VviLBDIf7* S-gene.

TATGGTTGTGCTGGTGCAATTAGTCACCTTCAGAAACAACTTAATGATCTCCAAGCAGAATTGGCTATGACACAAGCGGCGCTCCTTCTCATACAATGCCAACAACATGAACAAAATCATGAGAATACAGCCATCTTTGATCAGGACAAATATTTCAGCTCCGTCTTGGAGCCTTCTTGGCAATGAAGAAATCTCAGGGAATTAATTGGGAAGTTCAAATAAATTCTAGGTGGGTGGGTATCAGGATTAGTAATGCAAATGAGAGAAGATGCTAGCTTGATGTTCTATGTCATATCAATGATTTAGATCTTGAAAATCGAAATGAATTTGTATGATAGCTTCTTCCTAGCTAGTTGAATAAGTCTAGGCGGCGAAGGTCCAAGACTAATAGAAGAAAAAGGAGAGCCGTGTG
