# Supplementary figures and images for: RNAi of a Putative Grapevine Susceptibility Gene as a Possible Downy Mildew Control Strategy
Source: Front Plant Sci. 2021 May 28;12:667319. doi: 10.3389/fpls.2021.667319 (PMC8196239; doi:10.3389/fpls.2021.667319)

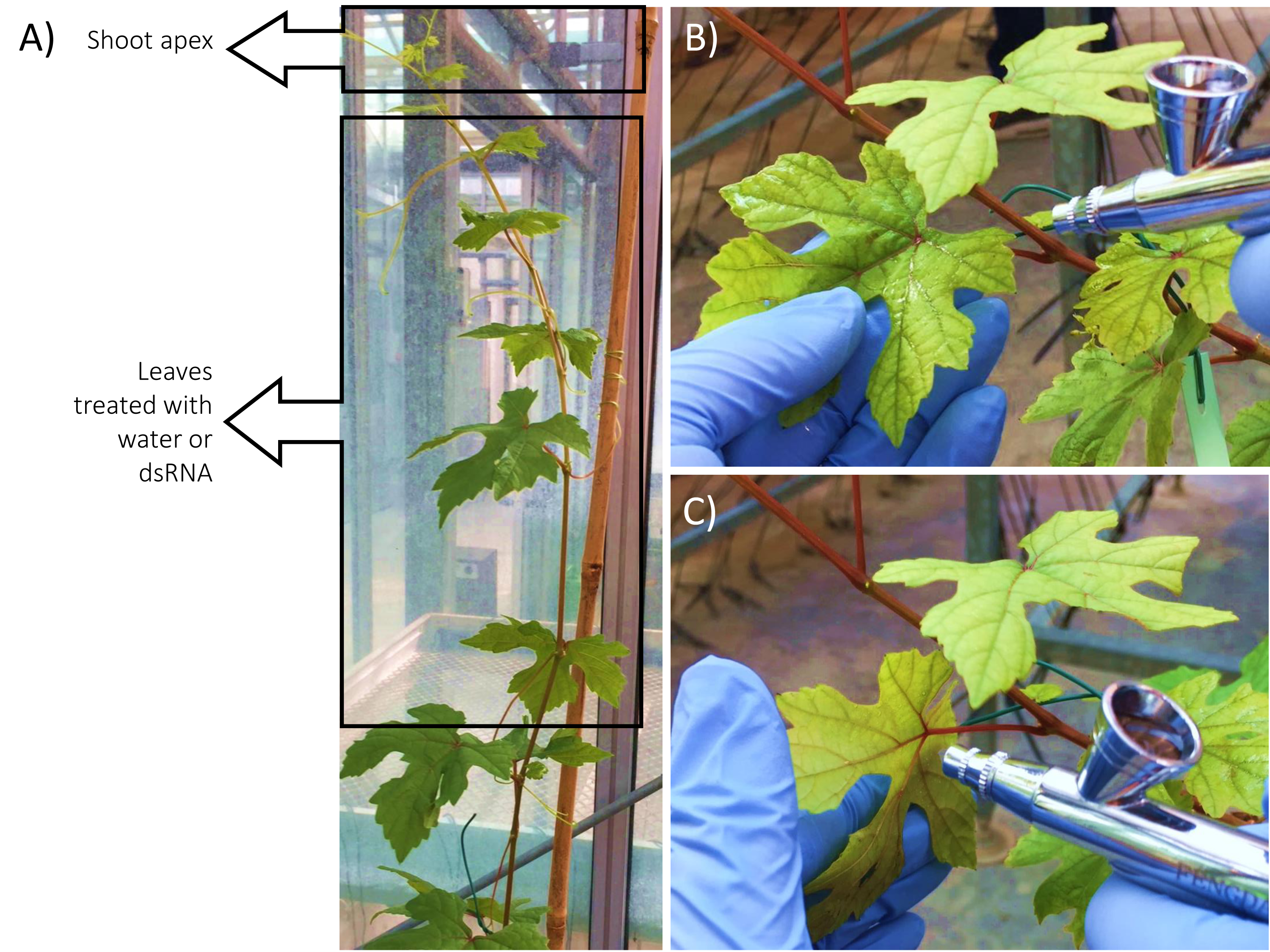

Supplement: Supplementary file 3 [file Image_1.TIF]

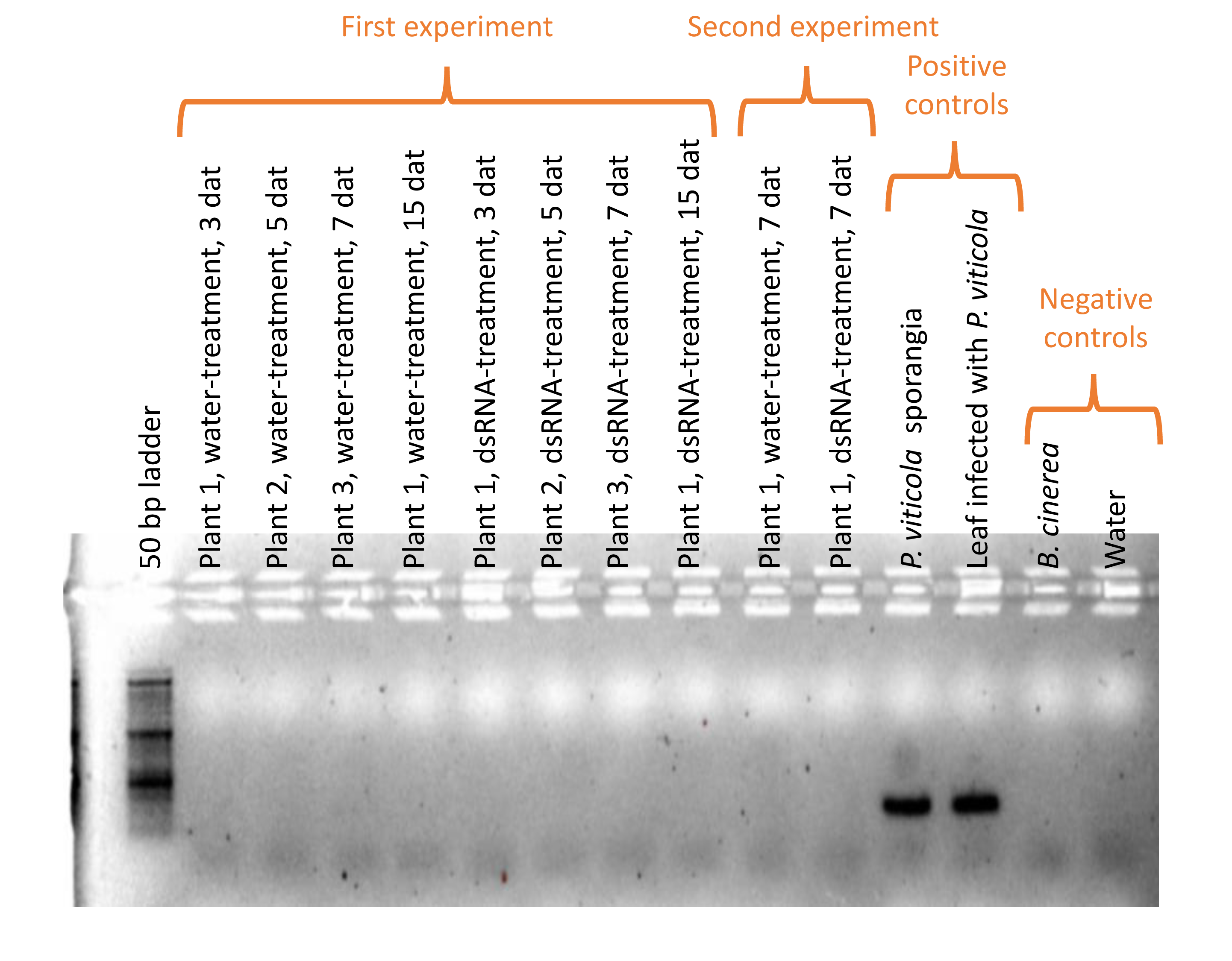

Supplement: Supplementary file 4 [file Image_2.TIF]
